# Pathologic Evaluation of Type 2 Porcine Reproductive and Respiratory Syndrome Virus Infection at the Maternal-Fetal Interface of Late Gestation Pregnant Gilts

**DOI:** 10.1371/journal.pone.0151198

**Published:** 2016-03-10

**Authors:** Predrag Novakovic, John C. S. Harding, Ahmad N. Al-Dissi, Andrea Ladinig, Susan E. Detmer

**Affiliations:** 1 Department of Veterinary Pathology, Western College of Veterinary Medicine, University of Saskatchewan, Saskatoon, SK, Canada; 2 Department of Large Animal Clinical Sciences, Western College of Veterinary Medicine, University of Saskatchewan, Saskatoon, SK, Canada; 3 University Clinic for Swine, Department for Farm Animals and Veterinary Public Health, University of Veterinary Medicine Vienna, Vienna, Austria; University of Hong Kong, CHINA

## Abstract

The pathogenesis of fetal death caused by porcine reproductive and respiratory syndrome virus (PRRSV) remains unclear. The objective of this study was to improve our understanding of the pathogenesis by assessing potential relationships between specific histopathological lesions and PRRSV RNA concentration in the fetuses and the maternal-fetal interface. Pregnant gilts were inoculated with PRRSV (n = 114) or sham inoculated (n = 19) at 85±1 days of gestation. Dams and their litters were humanely euthanized and necropsied 21 days later. PRRSV RNA concentration was measured by qRT-PCR in the maternal-fetal interface and fetal thymus (n = 1391). Presence of fetal lesions was positively related to PRRSV RNA concentration in the maternal-fetal interface and fetal thymus (*P*<0.05 for both), but not to the distribution or severity of vasculitis, or the severity of endometrial inflammation. The presence of fetal and umbilical lesions was associated with greater odds of meconium staining (*P*<0.05 for both). The distribution and severity of vasculitis in endometrium were not significantly related to PRRSV RNA concentration in maternal-fetal interface or fetal thymus. Endometrial inflammation severity was positively related to distribution and severity of vasculitis in endometrium (*P*<0.001 for both). Conclusions from this study suggest that type 2 PRRSV infection in pregnant gilts induces significant histopathological lesions at maternal-fetal interface, but they are not associated with presence of PRRSV in the maternal-fetal interface at 21 days post infection. Conversely, fetal pathological lesions are associated with presence of PRRSV in the maternal-fetal interface and fetal thymus, and meconium staining is significantly associated with the presence of both fetal and umbilical lesions observed 21 days post infection.

## Introduction

Porcine reproductive and respiratory syndrome virus (PRRSV) is one of the most important viral pathogens in swine production worldwide, causing one of the most costly diseases facing the North American swine industry with production losses estimated to be $664 million USD every year in the USA [1]. PRRSV is an enveloped, positive-stranded RNA virus, belonging to the genus Arterivirus, family Arteriviridae, in the order of Nidovirales [2,3]. All arteriviruses feature cytopathic replication in macrophages, the capacity to develop and preserve an asymptomatic infection, and cause severe and fatal disease [4]. Clinical presentation of the syndrome caused by PRRSV varies considerably between herds, ranging from asymptomatic to devastating disease [5]. The severity of PRRS clinical signs is influenced by virus strain involved, host immune status, genetic susceptibility [6], concurrent infections and other management factors [2]. Clinical disease in pregnant females is characterized by acute viremia and transplacental transmission in mainly third trimester resulting in reproductive failure [3,7].

In the reproductive form of PRRS in late gestation gilts or sows, gross and microscopic lesions are frequently located in the uterus, both in cases of natural and experimental PRRSV infection [8,9]. The myometrium and particularly endometrium are characterized by marked edema with lymphoplasmacytic and histiocytic endometritis with perivascular cuffing. Occasionally, segmental lymphoplasmacytic vasculitis and marked microseparations between endometrial epithelium and placental trophoblasts can be observed [3]. Infected sows and gilts farrow between 100–118 days of gestation with litters composed of variably sized normal, weak, and dead piglets that can be fresh stillborn (intrapartum death), autolytic, partially mummified or completely mummified [9–11]. Some dead and viable fetuses are covered with a brown mixture composed of meconium and amniotic fluid. Gross and microscopical lesions are mostly present in the umbilical cord and less often in the internal organs of fetuses [3]. Meconium staining of the fetuses indicates the occurrence of fetal stress during gestation, however,the specific pathogenic mechanism is unclear. Hypoxia was hypothesized to play an important role, but whether it is caused by disruption of the blood flow through the umbilical cord or due to lesions at the maternal-fetal interface still needs to be revealed [8].

Despite nearly 25 years of PRRS research, the primary mechanism of PRRSV-induced reproductive failure is poorly understood. The absence of significant microscopic lesions in the internal organs of stillborn piglets suggests that the fetal death might not be a sequela of PRRSV replication in the fetal tissues [12]. Recent studies propose that type-1 PRRSV replication within the fetal placental mesenchyme causing severe histopathological lesions at maternal-fetal interface in the third trimester of gestation is responsible for PRRSV-related reproductive disease [7]. One recent study with type 2 PRRSV infection found a lack of correlation between the presence of gross abnormalities in the fetus and productive fetal infection suggesting the source of reproductive pathology is potentially infection of tissues on the maternal side, damage to maternal tissues, or production of maternal factors that affect the fetus [13]. However, no published studies to date have investigated the relationship between the presence of the PRRSV in the uterus and the fetus and the severity and distribution of lesions at maternal-fetal interface, or evaluated potential involvement of maternal-fetal interface lesions in fetal death. The pathologic evaluation of a large dataset of maternal-fetal interface may cast some new information on the pathogenesis of transplacental PRRSV infection.

The objectives of this study were to: 1) qualitatively assess the PRRSV-associated microscopic lesions in the fetal tissues, uterus and placenta; 2) develop a grading scheme and assess the severity of microscopic lesions associated with PRRSV infection at maternal-fetal interface and fetuses; and 3) determine relationships between the severity and distribution of microscopic lesions, PRRSV RNA concentration in the maternal-fetal interface and the fetus, and fetal preservation status.

## Material and Methods

### Ethics statement

Inoculation of gilts or sows in the last trimester of gestation is a widely accepted and commonly used model for studying reproductive PRRS [12–17]. Although we recognize that some fetuses die after inoculation, death can occur unpredictably at any time after inoculation and no alternative models are available to study the reproductive effects of PRRSV infection. Monitoring fetal stress and discomfort is not feasible in a litter bearing species like swine. Dams were monitored daily according to a humane intervention point (HIP) checklist developed specifically for this project as is presented in detail (S1 File). Animal numbers were carefully considered and the number of inoculated gilts was selected to enable both deep phenotyping and genotyping of gilts and fetuses. Gilts were housed in Biosecurity Level 2 rooms, 3 to 5 animals per room, in 2' x 7' gestation pens on perforated flooring. Room temperature was maintained at 10−20^0^ C using mechanically controlled ventilation, and gilts were individually fed a non-medicated, commercial gestation diet once daily, and had *ad libitum* access to water. Given that fetal death was an outcome, the experimental protocol was considered carefully before approval by the University of Saskatchewan’s Animal Research Ethics Board. It adhered to the Canadian Council on Animal Care guidelines for humane animal use (permit #20110102).

### Experimental procedures

Detailed experimental protocol for this study has been published [18]. Briefly, on gestation day 85±1, 114 PRRSV-naïve pregnant gilts, approximately 10 months of age, were intramuscularly and intranasally inoculated with PRRSV (10^5^ TCID_50_ total dose, NVSL 97–7895, Gen Bank Accession No. AF325691) and 19 negative control pregnant gilts were similarly sham inoculated with the minimum essential medium. NVSL 97–7895 is highly abortifacient strain isolated from cases of severe reproductive failure by the Diagnostic Virology Unit of NVSL USDA/APHIS in a farm located in southeast Iowa in December 1996 [19]. The decision to use this particular strain in present study was based on the results of our pilot study during which NVSL 97–7895 demonstrated higher virulence than two other type 2 strains [20]. During the course of the present study, two gilts aborted one died, but no other gilts demonstrated severe clinically illness as previously described in detail [18]. At 21days post inoculation (DPI), dams and their litters were humanely euthanized for necropsy examination. Gilts were sedated with intravenous barbiturate (30 mL Euthanyl Forte supplying 16,200 mg pentobarbital sodium, Vetoquinol, Lavaltrie, QC) and humanely euthanized by cranial captive bolt followed by pithing. The gilt reproductive tract was removed intact and opened starting at the tip of each horn. A sample of the uterus with adherent placental layers (herein; maternal-fetal interface (MFI)), 10 cm in circumference and surrounding the umbilical attachment was collected from each fetus. From this sample, a full thickness, 4 cm^2^ section was collected in 10% buffered formalin for histology, and a 0.5 cm^2^ section was snap frozen in liquid nitrogen for PRRSV RNA quantification using an in-house quantitative real-time PCR assay (qRT-PCR) as previously described [18]. The preservation status was assigned for each fetus [18] based on the external gross appearance of their skin and umbilical cord as: viable (normal, white to purple skin with visible hair and regular umbilical cord), meconium-stained (skin covered with inspissated, brownish amniotic fluid and regular umbilical cord with edema), decomposed (<50% of skin discoloured, no blood in the umbilical cord), autolysed (>50% of skin discoloured), and mummified fetuses (dehydrated and small with crown-rump length <20 cm). Fetal samples of lung, liver, heart, thymus, mesenteric lymph node and cerebellum were collected in 10% buffered formalin for histology. A section of fetal thymus was also snap frozen in liquid nitrogen for PRRSV RNA quantification by real time qRT-PCR. Fetal samples for histopathology were not collected from autolyzed or mummified fetuses.

The formalin fixed tissues were processed within 48 hours of collection, paraffin embedded, and 4μm microsections were hematoxylin and eosin (HE) stained. Upon removal from liquid nitrogen, snap frozen gilt and fetal tissue samples were stored at -80°C pending RNA extraction from 10–20 mg using the RNeasy extraction kit (Qiagen Inc., Toronto, ON) according to the manufacturer’s instructions. PRRSV RNA concentration was measured using an in-house probe based real time qRT-PCR as previously described [18].

### Histopathology

Uterine and fetal samples (1452 infected and 231 negative controls) were assessed by pathologists (SED and PN) blinded to qRT-PCR results and inoculation status. Histopathological evaluation of the fetal organs, placenta, endometrium, myometrium, and umbilical cords was based on a qualitative (presence/absence) assessment of potentially significant pathological lesions associated with PRRSV. Assessment of fetal lesions included those previously described such as segmental lymphoplasmacytic arteritis and periarteritis in heart and lung [8], interstitial pneumonia [3], periportal hepatitis, lymphoplasmacytic myocarditis with the loss of myocardial fibers, and leukoencephalitis particularly affecting cerebellum [21]. Also, fetal thymuses and mesenteric lymph nodes were assessed for previously described lesions such as thymic cortical atrophy and lymph node necrosis, polycystic degeneration, polykaryocytes, germinal center hypertrophy, and hyperplasia [22,23]. Umbilical cords were evaluated for the presence of necrotizing umbilical arteritis and severe periarterial hemorrhage [8]. Uterine tissues with fetal placentas were assessed for previously described lesions in the myometrium, endometrium and placenta such as lymphohistiocytic vasculitis, endometritis, and placental microseparations [8,9].

### Histologic grading

A grading scheme was developed to score the endometrial inflammation, vasculitis severity, and vasculitis distribution. Only uterine tissue sections with fully attached fetal placenta were selected for grading of lesions (n = 679 from 110 infected gilts). Endometrial inflammation was scored by assessing the total area of the endometrium tissue present on the microscope slide at 200X field of magnification. The inflammation in the endometrium was categorized as follows: grade 0 (normal) = very rare inflammatory cells present; grade 1 (minimal) = inflammatory cells multifocally present in < 10% of the tissue section; grade 2 (mild) = multifocal to coalescing inflammatory cell infiltrate in 10–25% of the tissue; grade 3 (moderate) = diffuse inflammatory cell infiltrate in 25–50% of the tissue, and grade 4 (severe) = inflammatory cells diffusely present in >50% of the tissue section.

To score vasculitis severity, three 200X microscopic fields of the endometrium per uterine tissue section were selected randomly by relocating the slide to a new field of view whilst withdrawing eyes from the eyepiece (ocular lenses). Within each of those three fields, three blood vessels (at 3, 9 and 12 o’clock in the field) were scored at 400X magnification as: grade 1 = presence of inflammatory cells only within the blood vessel wall; grade 2 = presence of the inflammatory cells together with degeneration (vacuolation and splitting of smooth muscles) or necrosis of the blood vessel wall layers; grade 3 = presence of inflammatory cells together with degeneration and necrosis in the blood vessel wall layers. The nine individual vessel scores were averaged providing a single severity score per fetus.

The vasculitis distribution score was adapted from the previous work [24,25] and based on the distribution of vasculitis in the endometrium present in a 200X microscopic field as follows: grade 0 (normal) = no blood vessels affected; grade 1 = from 0 to <30% of vessels affected by vasculitis; grade 2 = from 30% to 70% of vessels affected by vasculitis and grade 3 = >70% of blood vessels affected by vasculitis in the tissue section.

### Statistical Analyses

All statistical analyses were performed using two-level, mixed-effects regression models that controlled for litter of origin as a random effect (Stata 13, StataCorp LP, TX, USA). A mixed effects logistic regression model (MELOGIT) was used to assess potential relationships between fetal lesions (presence/absence) and vasculitis distribution score, average vasculitis severity score, endometrial inflammation score, and PRRSV RNA concentration (logarithm_10_ target genomic copies/gram) in MFI and fetal thymus. MELOGIT was also used to assess potential relationships between fetal preservation status (viable/meconium-stained) and the presence of fetal lesions, umbilical lesions, distribution and severity of vasculitis and endometrial inflammation. Separate unconditional mixed effects linear regression models (MIXED) were used to assess potential relationships between average vasculitis severity score and PRRSV RNA concentration in the uterus or fetal thymus. For these models, average vasculitis severity score was natural log (ln) transformed to ensure the model assumptions of linearity and homogeneity were not violated. Separate unconditional, proportional odds models (MEOLOGIT) were used to assess potential relationships between vasculitis distribution score and PRRSV RNA concentration in the uterus or fetal thymus. MEOLOGIT models were also used to assess relationships between endothelial inflammation score and PRRSV RNA concentration in uterus or thymus. Potential violation of proportional odds assumption was evaluated by comparing the coefficients with those generated with an equivalent generalized ordered logistical (GOLR) model in which the RNA concentration variable was unconstrained. In all cases, the proportional odds assumptions were not violated (coefficients were similar in both the PO and GOLR models), so results of the proportional odds models are reported herein. Finally, the potential association between endothelial inflammation score and vasculitis distribution and severity was assessed in a single proportional odds model that contained both vasculitis variables as fixed effects. To visualize statistically significant relationships between dependent and independent variables in proportional odds models, probability plots (GLLAPRED) were generated. For all models, litter of origin was included as a random effect and all models were verified for normality and homogeniety of residuals. Statistical significance was assigned at the *P* < 0.05 level *a priori*.

## Results

Of the 114 gilts PRRSV-inoculated, two aborted, 1 died, and 1 had all uterine tissue samples with completely detached fetal placentas. All were removed from the analyses. There were 1452 fetuses from the remaining 110 PRRSV-infected gilts and 231 fetuses from the 19 control gilts (total = 1683). Of these, tissues were not collected from 585 autolysed and 35 mummified fetuses. Microscopic examination was performed on all available fetuses including 1069 lungs, 1077 livers, 1080 hearts, 1037 thymuses, 983 mesenteric lymph nodes and 1072 cerebella (Table 1). Some organs from some fetuses were inadvertently not collected.

**Table 1:** Numbers of fetal tissues with histopathological lesions in type 2 PRRSV-infected and negative control pregnant gilts inoculated at gestation day 85 (± 1d).

Fetal tissues							
	Lung	Liver	Heart	Thymus	Mesenteric Lymph Node	Cerebellum	Umbilical cord
Negative control	0/221*	0/223	0/224	0/222	0/204	0/224	1/228
PRRSV-infected	11/848	21/854	27/856	6/815	89/779	19/848	78/1321

* Number of tissues with lesions/number of tissues examined.

### Histopathology of the fetus

The most prevalent lesions in fetuses from infected gilts were observed in mesenteric lymph nodes and umbilical cords. In 11.4% of mesenteric lymph nodes, there was mild to moderate follicular atrophy with the replacement of lymphocytes by macrophages. In one fetus, multinucleated cells with cytological features resembling polykaryocytes were found. Rarely, fetal mesenteric lymph nodes demonstrated focal areas of lymphoid hyperplasia. Lesions in 5.9% of umbilical cords were characterized by focal to multifocal, mild lymphocytic perivascular cuffing. Occasionally, mild to moderate hemorrhage surrounding the umbilical arteries and vein was observed. For one fetus, there was diffuse and severe hemorrhage that markedly distended umbilicus. The presence of inflammatory cells in vessel walls (vasculitis) of the umbilical arteries or veins was not observed. In 3.2% of fetal hearts, there was mild, focal to multifocal, lymphocytic myocarditis and perivascular cuffing. Occasionally, myocardiocyte degeneration and necrosis were observed. In 2.5% of fetal livers, small infiltrates of lymphocytes were focally present in the portal areas (mild periportal hepatitis). In 2.2% of fetal cerebella, there was perivascular cuffing and gliosis. In one fetus, there was severe meningoencephalitis. In 1.3% of fetal lungs there was multifocal, mild thickening of the alveolar septa by small numbers of macrophages and lymphocytes (mild interstitial pneumonia). In 0.7% fetal thymuses mild to moderate atrophy was observed. No microscopic evidence of vasculitis was seen in any fetal tissue and no microscopic lesions were found in tissues obtained from fetuses of negative control pregnant gilts.

### Histopathology of the maternal-fetal interface

The most prevalent lesion observed at the MFI was a lymphohistiocytic endometritis, ranging from mild to severe. This was observed in 99.6% of PRRSV-infected MFI tissue samples (uterus with fetal placenta) corresponding to each fetus. Less prevalent were lymphocytic myometritis in 58.5% and lymphohistiocytic placentitis in 6.6% of PRRSV-infected gilts (Table 2). Most of the samples of MFI from the negative control gilts had no lesions. However, mild lymphocytic inflammation was observed in one myometrium (adjacent viable fetus without lesions) and two endometria (adjacent one viable fetus without fetal lesions, and one autolysed fetus) out of 230 negative control fetuses examined (Table 2). Vasculitis in the endometrium was primarily observed within the small calibre vessels, but occasionally arteries and veins were affected. The lymphocytes were found in all layers of the blood vessels, along with various degrees of degeneration of the tunica intima and media. Fibrinoid vascular degeneration was rarely observed.

**Table 2:** Number of uterine and placental tissues demonstrating histopathological lesions in type 2 PRRSV-infected and negative control pregnant gilts inoculated at gestation day 85 (± 1d).

Uterus			
	Myometrium	Endometrium	Placenta
Negative control	1/230*	2/230	0/230
PRRSV-infected	822/1404	1399/1404	91/1360

* Number of tissues with lesions/number of tissues examined. One section of maternal fetal interface (uterus with adherent fetal trophoblast) was collected at the umbilical stump of each fetuses examined.

### Relationship between fetal lesions and PRRSV RNA concentration

The PRRSV RNA concentration (target copies/mg) was measured in fetal thymus and sections of MFI adjacent the umbilical stump of each fetus. Across all PRRSV-infected gilts and fetuses, the average PRRSV RNA concentration (log10 copies/mg) in the MFI and fetal thymus was 2.97 (±2.23) and 3.17 (±2.72), respectively. The proportion of the litter and MFI samples that tested PRRSV qRT-PCR positive were very similar (S1 Fig and S2 Fig). Of the 679 fetuses with fully attached fetal placenta that were used in the histological analyses below, the average PRRSV RNA concentration (log10 copies/mg) in MFI and fetal thymus was 3.32 (±2.36) and 3.05 (±3.22), respectively. One or more lesions were present in the tissues of 96 of 679 of fetuses (14%). Similarly, 95 of 679 fetuses (14%) were meconium stained in this dataset. The presence of fetal lesions was positively related to PRRSV RNA concentration in the maternal-fetal interface (odds ratio [OR], 1.4; 95% CI 1.2 to 1.6; *P*<0.001) as well as in the fetal thymus (OR, 1.3; 95% CI 1.2 to 1.4; *P*<0.001). There was no significant relationship between the presence of fetal lesions and vasculitis distribution, vasculitis severity, or endometrial inflammation severity. The presence of fetal and umbilical lesions significantly increased the odds of a fetus being meconium stained compared to being viable (OR 2.1, 95% CI 1.1 to 4.1, *P*<0.05 for fetal lesions; OR 6.9, 95% CI 3.2 to 14.9, *P*<0.001 for umbilical lesions). However, fetal preservation status was not associated with vasculitis distribution or severity, or endometrial inflammation severity.

### Assessment of endometrial inflammation and vasculitis and relationship to viral load

**Fig 1:**
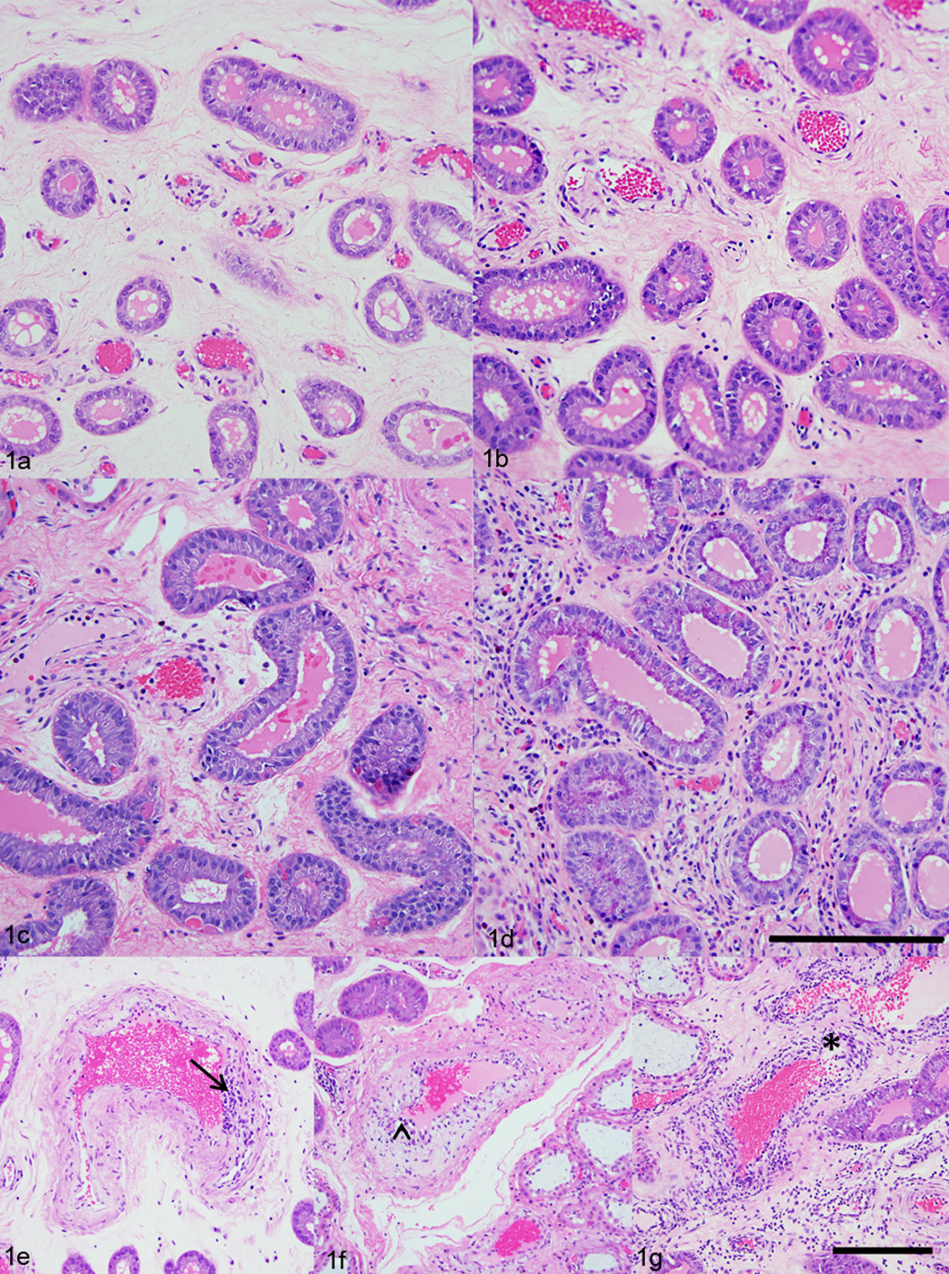
Histologic scores of endometrial inflammation and severity of vasculitis. (a) Uterus, endometrium, lamina propria; PRRSV-infected pregnant gilt; Hematoxylin and Eosin (HE). Minimal lymphohistiocytic endometritis (grade 1.). (b) Uterus, endometrium, lamina propria; PRRSV-infected pregnant gilt; HE. Mild lymphohistiocytic endometritis (grade 2.). (c) Uterus, endometrium, lamina propria; PRRSV-infected pregnant gilt; HE. Moderate lymphohistiocytic endometritis (grade 3.). (d) Uterus, endometrium, lamina propria; PRRSV-infected pregnant gilt; HE. Severe lymphohistiocytic endometritis (grade 4.) (Scale bar = 200 μm). (e) Uterus, endometrium, blood vessel; PRRSV-infected pregnant gilt; HE. Lymphocytic vasculitis (severity grade 1.) (arrow). (f) Uterus, endometrium, blood vessel; PRRSV-infected pregnant gilt; HE. Lymphocytic vasculitis with vacuolar degeneration of the cells in the *tunica intima* (severity grade 2.) (arrowhead). (g) Uterus, endometrium, blood vessel; PRRSV-infected pregnant gilt; HE. Severe lymphocytic vasculitis with necrosis (severity grade 3.) (asterisk) (Scale bar = 200 μm).

A total number of 679 uterine tissue sections from 110 PRRSV-infected pregnant gilts were scored for endometrial inflammation, vasculitis severity and vasculitis distribution (Fig 1). Results revealed that 72.9% of the uterine tissues demonstrated moderate (grade 3) lymphohistiocytic endometritis, while minimal evidence of lymphohistiocytic endometritis was found in only 2.5% tissue sections (grade 1). We found that in 67.3% of the tissue sections the vasculitis affected <30% of vessels (grade 1), and only in 2.4% of tissue sections, the vasculitis affected more than 70% of blood vessels (grade 3). Regarding the severity of the vasculitis in the endometrium, we found that 62.8% of tissue sections demonstrated grade 1, 33.1% grade 2, and 4.1% grade 3 severity score (Table 3). No evidence of vasculitis was found in 1.0% (7/679) of the endometrial tissues from PRRSV-infected pregnant gilts, and they were graded as normal (grade 0). Uterine tissue sections from the negative control gilts had no significant inflammatory lesions, and they were all graded as normal (grade 0).

**Table 3:** Numbers of uterine tissue sections scored for endometrial inflammation, distribution of vasculitis and severity of vasculitis distribution.

Uterine tissues					
	Grade 0	Grade 1	Grade 2	Grade 3	Grade 4
Endometritis inflammation score	0/679*	17/679	97/679	495/679	70/679
Vasculitis distribution score	7/679	457/679	199/679	16/679	0
Severity vasculitis score	157/679	269/679	225/679	28/679	0

* Number of tissues with score/number of tissues examined.

**Fig 2:**
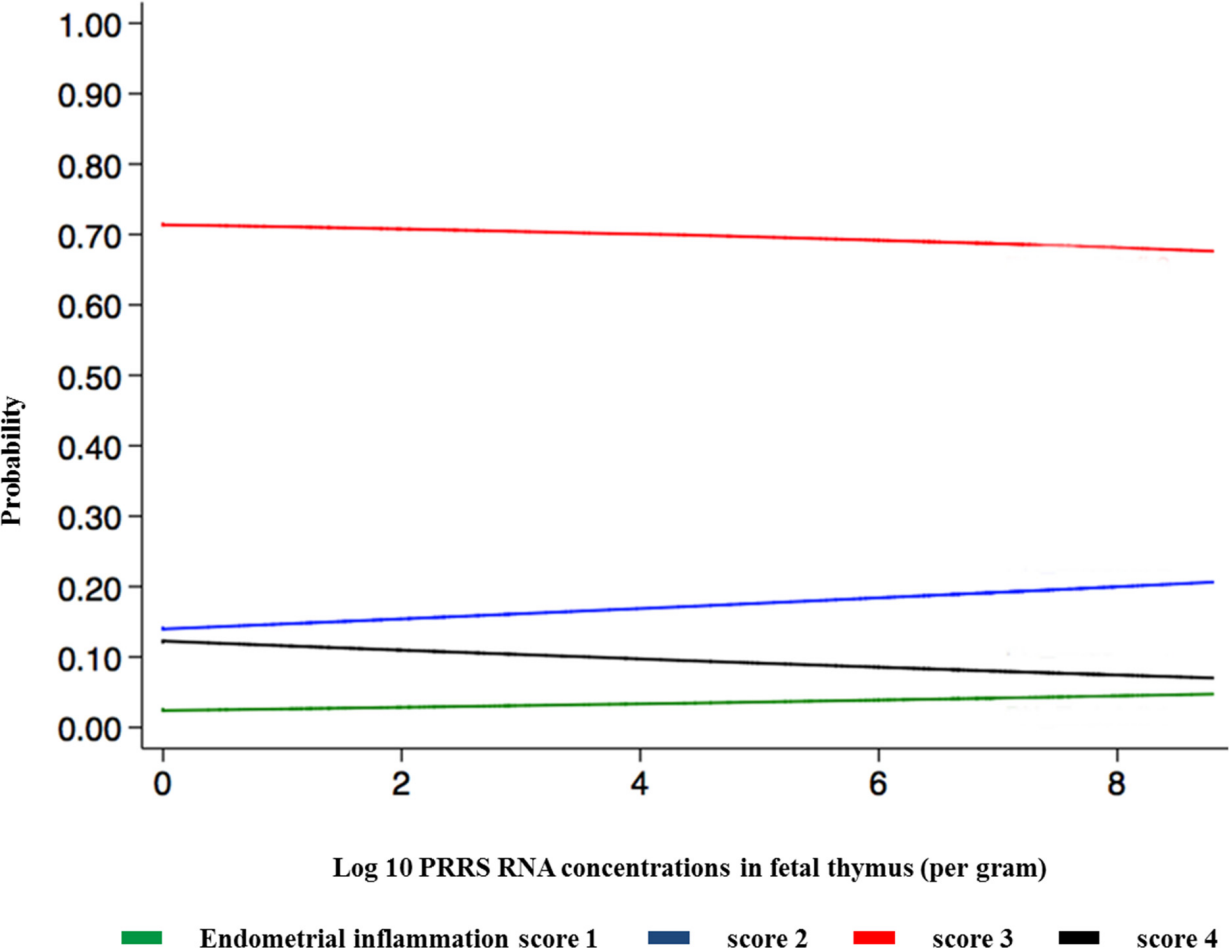
Relationship between endometrial inflammation and PRRSV RNA concentration (log_10_/gram) in fetal thymus. Endometrial inflammation severity is indicated by 4 coloured lines: minimal (green) = inflammatory cells multifocally present in < 10% of the tissue section; mild (blue) = multifocal to coalescing inflammatory cells infiltrate in 10–25% of the tissue; moderate (red) = diffuse inflammatory cell infiltrate in 25–50% of the tissue; severe (black) = inflammatory cells diffusely present in >50% of the tissue section. Results indicate increased viral load in fetal thymus is associated with decreased probability of moderate and severe endometrial inflammation observed at 21 dpi.

**Fig 3:**
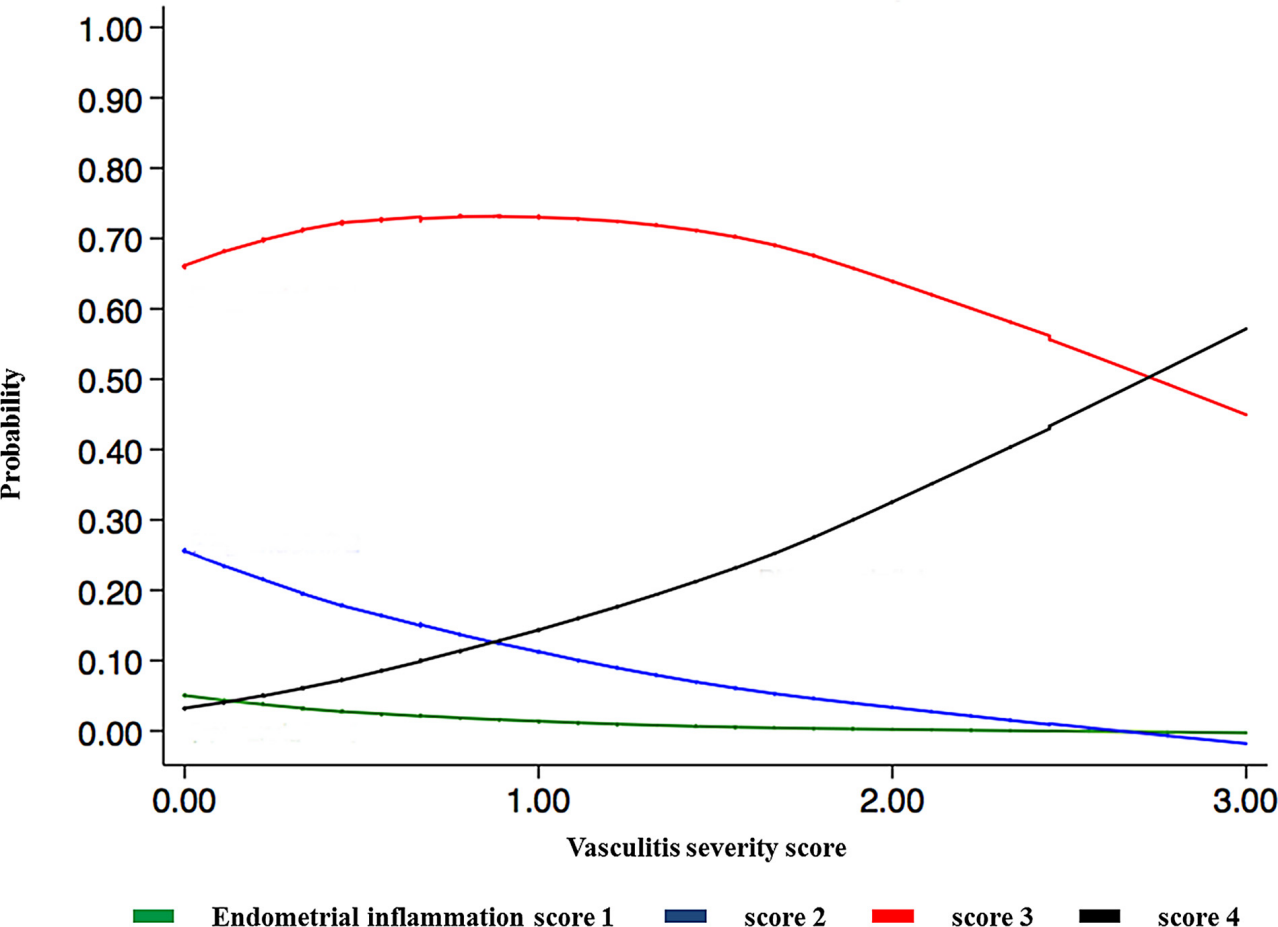
Relationship between endometrial inflammation and severity of vasculitis. Endometrial inflammation severity is indicated by 4 coloured lines: minimal (green) = inflammatory cells multifocally present in < 10% of the tissue section; mild (blue) = multifocal to coalescing inflammatory cells infiltrate in 10–25% of the tissue; moderate (red) = diffuse inflammatory cell infiltrate in 25–50% of the tissue; severe (black) = inflammatory cells diffusely present in >50% of the tissue section. Results indicate the probability of observing severe endometrial inflammation at 21 dpi increases with vasculitis severity.

**Fig 4:**
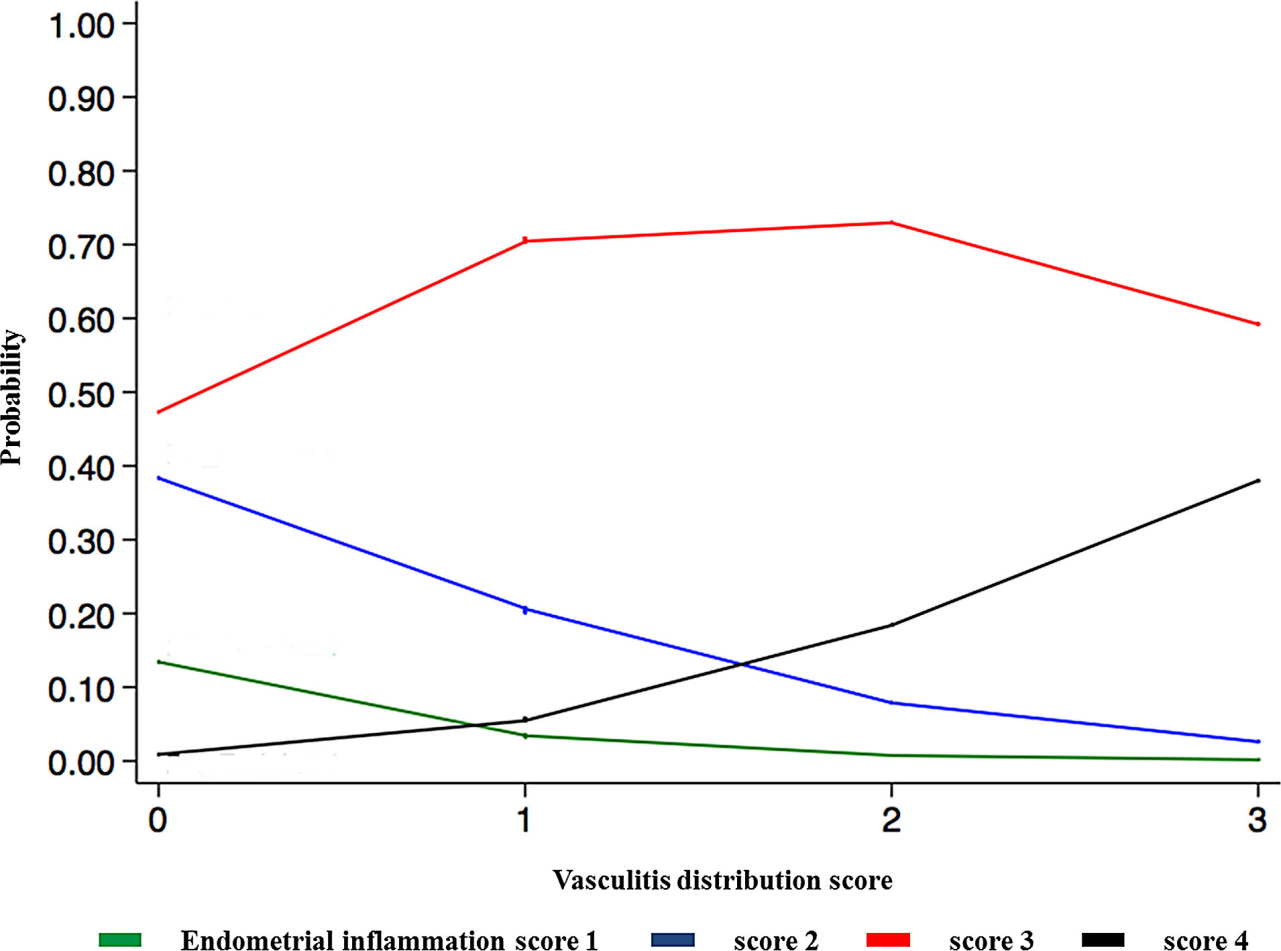
Relationship between endometrial inflammation and distribution of vasculitis. Endometrial inflammation severity is indicated by 4 coloured lines: minimal (green) = inflammatory cells multifocally present in < 10% of the tissue section; mild (blue) = multifocal to coalescing inflammatory cells infiltrate in 10–25% of the tissue; moderate (red) = diffuse inflammatory cell infiltrate in 25–50% of the tissue; severe (black) = inflammatory cells diffusely present in >50% of the tissue section. Results indicate the probability of observing severe endometrial inflammation at 21 dpi increases with more extensive vasculitis distribution.

There was no significant relationship between endometrial inflammation score and PRRSV RNA concentration in the uterus, and neither vasculitis distribution nor average vasculitis severity score was significantly related to PRRSV RNA concentration in the uterus or in fetal thymus. Endometrial inflammation severity was negatively related to PRRSV RNA concentration in the fetal thymus (*P*<0.05; Fig 2). Endometrial inflammation score was positively related to both vasculitis distribution (*P*<0.001) and severity of vasculitis in the endometrium (*P*<0.001; Fig 3 and Fig 4).

## Discussion

The present study is part of a large scale reproductive PRRS project investigating the phenotypic and genotypic factors associated with viral load and fetal death. The decision to inoculate at 85 days of gestation was based on the results of previous studies that confirm that late gestation infection between 72 to 93 days consistently results in transplacental infection and reproductive failure that is similar to field observations[7]. While the decision to terminate at 21 dpi was potentially past peak viral load and acute-stage uterine histopathology, it was required to fulfill the overarching project objectives including the identification of factors associated with fetal death and viral load, and to advance the understanding of factors involved in the pathogenesis of fetal death. In spite of the single collection time point well past the acute-stage in the dam, the present histopathological study enabled the unprecedented assessment of a large number of fetal and maternal tissues, and determined potential relationships amongst lesions and viral load.

The objectives of this present study were to evaluate the microscopic lesions in the uterus, placenta, and fetal tissues, histologically grade lesion severity associated with PRRSV infection at the maternal-fetal interface, and determine potential associations between severity of lesions at maternal-fetal interface, fetal lesions, PRRSV viral load in the uterine tissue and the fetus, and fetal preservation status.

The most prominent and consistent lesions were observed in the uterus and the fetal placenta while fetal histopathological lesions were less frequent and more variable in severity and histological presentation. The majority of uteri and fetal placental samples obtained from PRRSV-infected pregnant gilts demonstrated significant inflammatory lesions affecting mostly endometrium, fetal placenta, and blood vessels. Furthermore, when severity and distribution of inflammation lesions were histologically graded the majority of uterine tissue samples exhibited moderate (73%) or severe (10%) lymphohistiocytic endometritis, and almost one-third of samples demonstrated severe vasculitis in more than 30% of blood vessels. These findings indicate that inflammation in the uterus, fetal placenta, and endometrial blood vessels are the most prominent pathological characteristics of type 2 PRRSV infection in pregnant gilts at 21 days post inoculation. This finding is also in agreement with previously published studies [8]. In the present study, we found a significant relationship between the distribution and the severity of the vasculitis in the lamina propria, and the severity of endometrial inflammation in the uterus. This finding supports the important role of endometrial blood vessels in PRRSV transmission into the uterus of viremic pregnant gilts. The exact mechanism of transplacental viral transmission is still unknown, but it has been hypothesized that it is mediated either by the infection of blood monocytes [7] and/or endothelial cell of the endometrial blood vessels [26]. However, there was no significant relationship between the concentration of PRRSV RNA in the maternal-fetal interface and the severity of endometrial inflammation or the distribution and severity of endometrial vasculitis. Also, no significant association was found between the concentration of PRRSV RNA in the fetal thymus and the distribution and severity of endometrial vasculitis. A statistically significant negative relationship between endometrial inflammation and PRRSV RNA concentration in fetal thymus was, in our opinion, most likely related to collecting samples at 21 dpi in our study. It is known that viremia in the sows and postnatally infected pigs reaches peak at 7–9 days post inoculation. However, the duration of viremia and presence of PRRSV in the fetus or congenitally infected pigs is slightly longer [10]. The reason for the longer viremia in the fetus is due to PRRSV replication in the primary lymphoid organs such as thymus [15]. It is possible that the severity of endometrial inflammation in some infected gilts had started to subside before 21 dpi, while PRRSV in fetus continued to replicate unabated until termination. However, the statistically significant relationship between PRRSV RNA concentration in the fetal thymus and the severity of endometrial inflammation found in this study (Fig 2) may be too weak to be biologically relevant based on the value of the regression coefficient (β = 0.08).

The most important finding in this study was a significant, positive relationship between the presence of fetal lesions and PRRSV RNA concentration, both in the MFI and the fetal thymus. In other words, the odds of a fetus having PRRSV-associated lesions increased as did PRRSV viral load in both tissues. Even more, meconium staining, an early sign of fetal compromise, was significantly associated with the presence of histological fetal and umbilical lesions. In this study, we determined that fetuses with umbilical lesions were seven times more likely to be meconium stained than those fetuses without lesional cords. Fetuses with lymphoid and/or systemic lesions were two times more likely to be meconium stained than fetuses without these lesions. These results revealed that not only umbilical lesions as previously suspected [8] but also fetal lesions, play a significant role in the pathogenesis of fetal demise following type 2 *in utero* PRRSV infection.

In retrospect, the ability to quantify viral load in the maternal (endometrium) and fetal (adherent placenta) portions of the MFI would have been very beneficial, but it was not possible based on the volume of samples processed on a given sample collection day. That being said, pathological processes observed at 21 dpi at the MFI have no significant relationship on the occurrence of fetal lesions or fetal preservation status, likely because the inflammatory process is well past its peak by this time. As these results are in contradiction with some previous reports [7,12] that suggested that fetal death in the reproductive PRRSV infection is primarily a consequence of the pathological processes at the MFI and not in the fetus itself, we have additional experiments planned using early termination points to help clarify this issue. However, our present results indicate that pathological processes associated with fetuses are essential predictors of the fetal preservation status. This finding is in accordance with some of the most recent studies indicating that presence of PRRSV in fetuses, particularly in the thymus, increased the likelihood of fetal death [27].

## Conclusions

This large-scale, multicenter, challenge experiment enabled an extensive evalaution of fetal and uterine lesions caused by type 2 PRRSV infection in third trimester pregnant gilts at 21 dpi, and provided new insights into the pathogenesis of fetal death. Severe microscopic lesions were observed in the uterus and fetal placenta, but not in the fetus. Lymphohistiocytic endometritis and lymphocytic myometritis were observed in nearly 100% and 60% of uterine samples, respectively, whereas the most common fetal lesions, follicular atrophy of mesenteric lymph nodes and lymphocytic perivascular cuffing in umbilical vessels, were markedly less prevalent. The presence of fetal lesions was positively associated with PRRSV viral load in uterus and fetal thymus, but not with severity of uterine pathology. Moreover, the presence of fetal and umbilical lesions, but not uterine pathology, increased the likelihood of fetal meconium staining. While the distribution and severity of inflammation in endometrial blood vessels were positively associated with inflammation in the lamina propria, the severity and distribution of vasculitis, and severity of endometrial inflammation were not associated with PRRSV RNA concentration at the maternal-fetal interface or in fetal thymus at 21 dpi. The overall conclusion of this study is that although type 2 PRRSV infection in pregnant gilts induces significant pathological lesions at the maternal-fetal interface, fetal and umbilical pathology and PRRSV viral load all likley contribute to fetal compromise and death.

## Supporting Information

S1 Fig Proportion of litter tested PRRSV qRT-PCR positive. Frequency distribution of the percentages of fetuses within litters that tested positive for PRRSV using qRT-PCR at 21 days post-inoculation at gestation day 85 in PRRSV infected pregnant gilts.
(TIF)

S2 Fig Proportion of maternal-fetal interface samples tested PRRSV qRT-PCR positive. Frequency distribution of the within litter percentages of maternal-fetal interface samples corresponding to each fetus that tested positive for PRRSV using qRT-PCR at 21 days post-inoculation at gestation day 85 in PRRSV infected pregnant gilts.
(TIF)

S1 File Humane Intervention Point Checklist. (PDF)
