# Supplementary figures and images for: Pathologic Evaluation of Type 2 Porcine Reproductive and Respiratory Syndrome Virus Infection at the Maternal-Fetal Interface of Late Gestation Pregnant Gilts
Source: PLoS One. 2016 Mar 10;11(3):e0151198. doi: 10.1371/journal.pone.0151198 (PMC4786155; doi:10.1371/journal.pone.0151198)

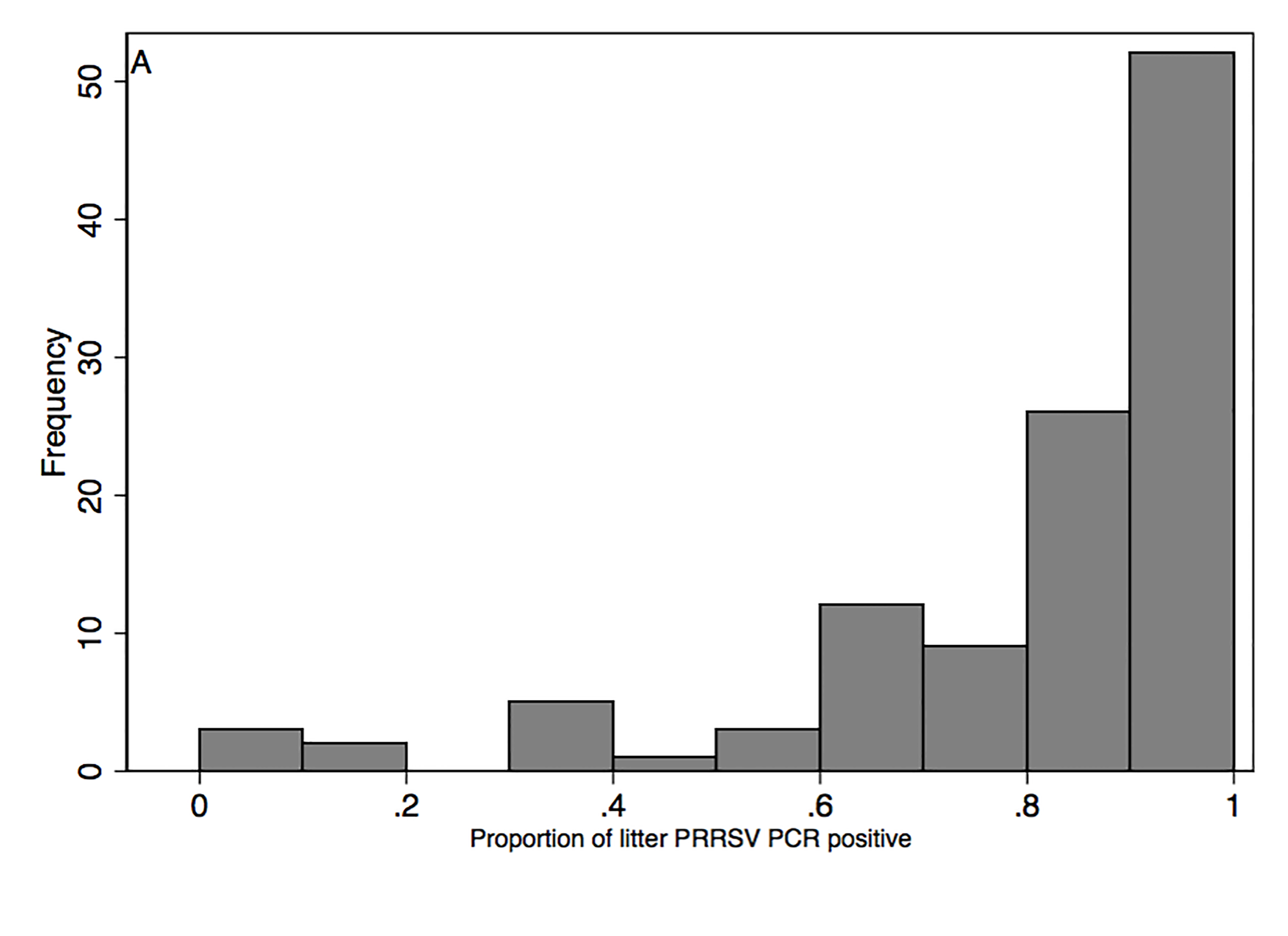

Supplement: S1 Fig — Frequency distribution of the percentages of fetuses within litters that tested positive for PRRSV using qRT-PCR at 21 days post-inoculation at gestation day 85 in PRRSV infected pregnant gilts. (TIF) [file pone.0151198.s001.tif]

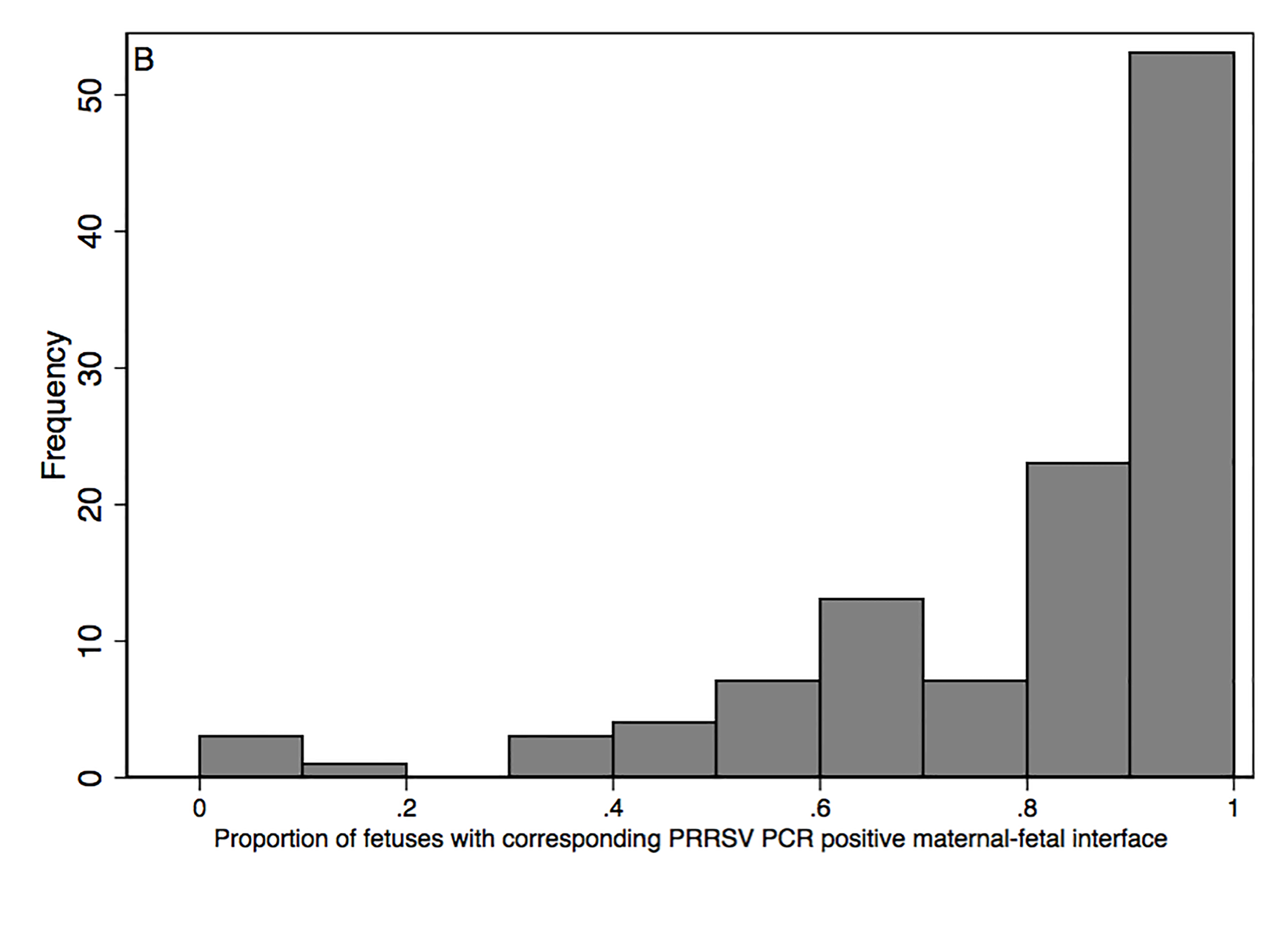

Supplement: S2 Fig — Frequency distribution of the within litter percentages of maternal-fetal interface samples corresponding to each fetus that tested positive for PRRSV using qRT-PCR at 21 days post-inoculation at gestation day 85 in PRRSV infected pregnant gilts. (TIF) [file pone.0151198.s002.tif]
