# Supplementary material for: Pathologic Evaluation of Type 2 Porcine Reproductive and Respiratory Syndrome Virus Infection at the Maternal-Fetal Interface of Late Gestation Pregnant Gilts
Source: PLoS One. 2016 Mar 10;11(3):e0151198. doi: 10.1371/journal.pone.0151198 (PMC4786155; doi:10.1371/journal.pone.0151198)
Supplement: S1 File — (PDF) [file pone.0151198.s003.pdf]

Humane Intervention Point (HIP) Checklist

Title: PRRSV Pregnant Gilt Model – University of Saskatchewan
Date submitted: Oct-2011
AREB_AUP #: 20110102

Pigs will be monitored twice daily in the pre-inoculation period. Following inoculation, all pigs including controls will be assessed at least twice daily for illness and the following HIP checklist completed. Rectal temperature will be taken daily for 5 days following inoculation, then 2-3 times per week thereafter.

1. **Animals with any the following clinical signs will be euthanized immediately. This requires verbal authorization from the principal investigator**.
   - Body temperature < 35 ^{o}C
   - Lateral recumbency and non-responsive
   - Severe dyspnea or respiratory distress (noted by obvious abdominal breathing)
   - Severe depression (moribund) accompanying abortion
   - Fracture, or injury that is life threatening or causing unrelenting pain or discomfort

Note: Gilts which abort will be euthanized at 21 DPI as planned.

2. **Clinical scoring system: Each pig will be observed each morning (before 10 am) and the clinical signs of disease will be assessed as follows:** Note: If any clinical sign scores >= 2, medical intervention may be warranted. Please consult the animal care veterinarian for treatments.

Score	Clinical Signs
**A: Body temperature**	
0	37-39.5^{o}C
1	39.6-40.5^{o}C or 36-36.9^{o}C
2	>40.5^{o}C or <36°C
**B: Responsiveness**	
0	Bright and alert
1	Depressed, reluctant to get up, but gets up following stimulation
2	Down, doesn't respond with stimulation or demonstrates seizures (leg paddling, recumbency, opisthotonous)
**C: Respiration**	
0	Normal
1	Panting, slight abdominal breathing (dyspnea) and/or coughing
2	Panting, marked abdominal breathing (dyspnea) and/or persistent paroxysmal coughing
**D: Appetite**	
0	Normal appetite
1	Reduced feed intake (does not eat all feed within 24 hours)
2	Anorexic (no feed consumption over 24 hours)
